# Autophagy Plays an Important Role in Anti-inflammatory Mechanisms Stimulated by Alpha7 Nicotinic Acetylcholine Receptor

**DOI:** 10.3389/fimmu.2017.00553

**Published:** 2017-05-16

**Authors:** Bo-Zong Shao, Ping Ke, Zhe-Qi Xu, Wei Wei, Ming-He Cheng, Bin-Ze Han, Xiong-Wen Chen, Ding-Feng Su, Chong Liu

**Affiliations:** ^1^Department of Pharmacology, Second Military Medical University, Shanghai, China; ^2^Institute of Quality and Standard for Agro-Products, Zhejiang Academy of Agricultural Sciences, Hangzhou, China; ^3^Cardiovascular Research Center, Temple University School of Medicine, Philadelphia, PA, USA

**Keywords:** alpha7 nicotinic acetylcholine receptor, autophagy, neuroinflammation, experimental autoimmune encephalomyelitis, microglia

## Abstract

Alpha7 nicotinic acetylcholine receptor (α7nAChR) has been reported to alleviate neuroinflammation. Here, we aimed to determine the role of autophagy in α7nAChR-mediated inhibition of neuroinflammation and its underlying mechanism. Experimental autoimmune encephalomyelitis (EAE) mice and lipopolysaccharide-stimulated BV2 microglia were used as *in vivo* and *in vitro* models of neuroinflammation, respectively. The severity of EAE was evaluated with neurological scoring. Autophagy-related proteins (Beclin 1, LC3-II/I, p62/SQSTM1) were detected by immunoblot. Autophagosomes were observed using transmission electron microscopy and tandem fluorescent mRFP-GFP-LC3 plasmid was applied to test autophagy flux. The mRNA levels of interleukin-6 (IL-6), IL-1β, IL-18, and tumor necrosis factor-α (TNF-α) were detected by real-time PCR. We used 3-methyladenine (3-MA) and autophagy-related gene 5 small interfering RNA (*Atg5* siRNA) to block autophagy *in vivo* and *in vitro*, respectively. Activating α7nAChR with PNU282987 ameliorates EAE severity and spinal inflammatory infiltration in EAE mice. PNU282987 treatment also enhanced monocyte/microglia autophagy (Beclin 1, LC3-II/I ratio, p62/SQSTM1, colocalization of CD45- or CD68-positive cells with LC3) both in spinal cord and spleen from EAE mice. The beneficial effects of PNU282987 on EAE mice were partly abolished by 3-MA, an autophagy inhibitor. *In vitro*, PNU282987 treatment increased autophagy and promoted autophagy flux. Blockade of autophagy by *Atg5* siRNA or bafilomycin A1 attenuated the inhibitory effect of PNU282987 on IL-6, IL-1β, IL-18, and TNF-α mRNA. Our results demonstrate for the first time that activating α7nAChR enhances monocyte/microglia autophagy, which suppresses neuroinflammation and thus plays an alleviative role in EAE.

## Introduction

It is commonly believed that microglia contribute to the triggering of inflammatory responses in central nervous system (CNS), mainly through the induction of inflammatory cytokine production as well as the immune responses (1, 2). However, the overactivation of microglia may lead to the pathogenesis and aggravation of CNS damage in inflammatory diseases including multiple sclerosis (MS) (3–5). MS is recognized as a chronic inflammatory autoimmune disorder featuring CNS demyelination and neurodegeneration (6, 7). Accumulation and overactivation of microglia greatly aggravates the severity of symptoms and demyelination in experimental autoimmune encephalomyelitis (EAE) mice (8, 9). Thus, suppressing microglia-mediated inflammation serves as a potential therapeutic strategy for MS or EAE.

Alpha7 nicotinic acetylcholine receptor (α7nAChR) is a subtype of nAChRs, which is a member of superfamily of cys-loop cationic ligand-gated channels (10, 11). Studies from our lab previously showed that α7nAChR was associated with various cardiovascular diseases (12, 13). Recently, there is evidence that activation of α7nAChR contributes to the alleviation of neuroinflammation in EAE model (14–16), but the underlying mechanism has not been fully clarified.

Autophagy is a self-protecting cellular catabolic pathway relying on lysosomes. Some long-lived proteins, as well as damaged organelles and misfolded proteins are degraded and recycled through autophagy process (17–19). It is widely acknowledged that autophagy is closely associated with CNS disorders, including cerebral ischemia, Parkinson’s disease, and MS (20–23). It has been reported that inducing autophagy could ameliorate several neurodegenerative diseases (24, 25). Studies demonstrated that inducing autophagy could inhibit inflammation, especially in inflammatory or immune cells such as macrophages and dendritic cells (26, 27). Recently, it has been reported that enhancement of autophagy could ameliorate the pathogenesis of MS or EAE disease through the limit of inflammation (21, 28, 29). However, whether autophagy plays a role in α7nAChR-mediated alleviation of neuroinflammation remains unclear.

In this study, we raised the hypothesis that activation of α7nAChR could promote monocyte/microglia autophagy, which inhibited the production of inflammatory cytokine and thus contributed to the attenuation of EAE severity. Our study may provide a novel therapeutic strategy for the treatment of MS.

## Materials and Methods

### Animal Care and Use

C57BL/6J mice (8–10 weeks old, male) were provided by Shanghai Super-B&K Laboratory Animal Corp. Ltd. (Shanghai, China). Mice were separately housed and had free access to water and standard chow diet (Shanghai Super-B&K Laboratory Animal Corp. Ltd., Shanghai, China). Experimental mice were maintained in specific pathogen-free conditions under a 12/12 h light cycle (on: 8:00 am) at 23 ± 2°C and 60 ± 10% humidity.

### BV2 Microglia Culture and Treatment

Murine BV2 microglia were provided by Shanghai Jining Corp. Ltd. (Shanghai, China). Cells were cultured with DMEM (Gibco, Grand Island, NY, USA) supplemented with 10% (vol/vol) fetal bovine serum (FBS) (Gibco, Grand Island, NY, USA) at 37°C in a humidified incubator with 5% CO_2_. BV2 microglia were challenged with lipopolysaccharide (LPS) (Sigma-Aldrich, St. Louis, MO, USA) in a dose of 100 ng/ml with or without 0.1, 1, and 10 µM PNU282987 (Sigma-Aldrich, St. Louis, MO, USA) for 12 h. In another set of experiments, BV2 microglia were preincubated with compound C (10 µM, Sigma-Aldrich, St. Louis, MO, USA), an AMPK inhibitor, for 10 min before LPS and PNU282987 treatment. For the blockade of autophagy *in vitro*, bafilomycin A1 (5 nM, Selleckchem, Houston, TX, USA) or autophagy-related gene 5 small-interfering RNA (*Atg5* siRNA) were applied for study.

### Transient Transfection of *Atg5* siRNA

The following siRNAs against *Atg*5 (Gene ID: 11793) were synthesized by Genepharm Biotech (Shanghai, China): siRNA1, 5′-CUCUCUAUCAGGAUGAUTT-3′, 5′-AUCUCAUCCUGAUAGAGAGTT-3′; siRNA2, 5′-GACGUUGGUAACUGACAAATT-3′, 5′-UUUGUCAGUUACCAACGUCTT-3′; siRNA3, 5′-GCGGUUGAGGCUCACUUUATT-3′, 5′-UAAAGUGAGCCUCAACCGCTT-3′; siRNA4, 5′-GCUACCCAGAUAACUUUCUTT-3′, 5′-AGAAAGUUAUCUGGGUAGCTT-3′. All of the four siRNAs comprised 21 nucleotides and contained symmetric 3′ overhangs of two deoxythymidines. BV2 microglia were transfected with *Atg5* siRNA as described before (30, 31). In brief, murine BV2 microglia were cultured with DMEM supplemented with 10% (vol/vol) FBS and grown to 30–50% confluency in six-well plates for transfection. The control or *Atg*5 siRNA (5 nM) was transfected into cells using siRNA-Mate (Genepharm) reagent in the form of siRNA–siRNA-Mate complex. After 24 h incubation, the medium was changed to fresh 10% FBS-loaded DMEM for the analysis of protein knockdown or further experiments.

### EAE Induction and Assessment

Mice were carefully grouped and selected randomly for studies. During animal experiments, a design of single-blind study was applied. EAE was induced in mice (8–10 weeks old, male) as previously reported (32, 33). In brief, mice were subcutaneously immunized with 200 µg MOG_35-55_ in Complete Freund’s adjuvant (Sigma-Aldrich, St. Louis, MO, USA) contained with heat-killed *Mycobacterium tuberculosis* (H37RA strain, 5 mg/ml) (BD Diagnostics, Franklin Lakes, NJ, USA). Pertussis toxin (Calbiochem, Billerica, MA, USA) in a dose of 200 ng for each mouse was injected on days 0 and 2 *via* i.p. Experimental mice were examined and evaluated every day for clinical signs and were scored according to the following criteria: “0,” no clinical signs; “1,” paralyzed tail; “2,” paresis; “3,” paraplegia; “4,” paraplegia with forelimb weakness or paralysis; “5,” moribund or death. For the treatment of drugs, PNU282987 (0.1 mg/kg, i.p.) or 3-methyladenine (3-MA) (10 mg/kg, i.p.) (Sigma-Aldrich, St. Louis, MO, USA) was injected once a day from day 3 till the end of the study. Mice were treated with saline as vehicle control (100 µl for each mouse).

### Histopathological Analysis

Experimental mice were anesthetized with phenobarbital sodium (35 mg/kg, i.p.) and sacrificed by cervical dislocation. Animals were then perfused with PBS (pH 7.4, 20 ml) followed by 4% (w/v) paraformaldehyde (20 ml). Spinal cords were subsequently excised and further fixed in 4% (w/v) paraformaldehyde overnight. Five-μm-thick sections of spinal cords embedded with paraffin were stained by hematoxylin and eosin as well as luxol fast blue to measure spinal inflammatory infiltration or demyelination, respectively.

### Immunofluorescence Microscopy

Immunofluorescence microscopy of paraffin-embedded sections of spinal cords was performed. In brief, 5-μm-thick paraffin-embedded sections of spinal cords were deparaffinized with xylene, rehydrated, blocked with normal goat serum, and incubated with one of the following antibodies: microtubule associated protein 1 light chain (LC) 3 (1:200, Novus Biologicals, Littleton, CO, USA), CD45 (1:200, Abcam, Cambridge, MA, USA), CD68 (1:200, Goodbio, Wuhan, Hubei, China). After extensive washing, the sections were incubated with double immunofluorescent staining including Alexa-488 and Alexa-647-labeled secondary antibodies (1:500, Invitrogen, USA) incubation for 1 h at room temperature. After being washed, slides were mounted with Vectashield mounting medium containing DAPI (Vector Laboratories, Burlingame, CA, USA) and the colocalization was detected by means of a confocal laser scanning microscope (Fluoview FV1000; Olympus, Tokyo, Japan).

### Immunoblot Analysis

BV2 microglia or spinal cord and spleen from EAE mice were washed with PBS for one time and lysed in lysis buffer on ice for 30 s. Protein concentration was detected by the bicinchoninic acid method (Thermo Scientific, Pittsburgh, PA, USA). Samples were loaded in 10% or 12% Tris/Gly gels, subjected to SDS-PAGE, and transferred on NC membranes (Millipore, Billerica, MA, USA). Immunoblot was conducted using the rabbit anti-Beclin 1 monoclonal antibody (1:500; Cell Signaling Technology, Danvers, MA, USA), rabbit anti-LC3 polyclonal antibody (1:500; Novus Biologicals, Littleton, CO, USA), rabbit anti-p62 antibody (Cell Signaling Technology, Danvers, MA, USA), rabbit anti-adenosine 5’-monophosphate (AMP)-activated protein kinase (AMPK) antibody (1:500, Cell Signaling Technology, Danvers, MA, USA), rabbit anti-phosphorylated AMPK antibody (1:500, Cell Signaling Technology, Danvers, MA, USA) anti-mammalian target of rapamycin rabbit (mTOR) antibody (1:500, Cell Signaling Technology, Danvers, MA, USA), rabbit anti-phosphorylated mTOR antibody (1:500, Cell Signaling Technology, Danvers, MA, USA), rabbit anti-p70 ribosomal protein S6 kinase (p70S6K) antibody (1:500, Cell Signaling Technology, Danvers, MA, USA), rabbit anti-phosphorylated p70S6K antibody (1:500, Cell Signaling Technology, Danvers, MA, USA) and mouse anti-glyceraldehyde-3-phosphate dehydrogenase (GAPDH) antibody (1:1000, Beyotime Biotechnology, Shanghai, China). After that, the membranes were incubated with a Donkey anti-Rabbit or Donkey anti-mouse secondary antibody (1:5,000, LI-COR Biosciences, Lincoln, NE, USA) accordingly. Images were obtained and analyzed using the Odyssey infrared imaging system (LI-COR Bioscience, Lincolin, NE, USA).

### Reverse Transcription and Real-time PCR

TRIzol reagent (Invitrogen, Carlsbad, CA, USA) was used for the extraction of total RNA from BV2 microglia. Reverse transcription was conducted for the extracted RNA to obtain the cDNA with PrimeScript™ RT Master Mix (Takara, Otsu, Shiga, Japan). Real-time PCR was then conducted in the LightCycler quantitative PCR apparatus (Stratagene, Santa Clara, CA, USA) using the FastStart Unitversal SYBR Green Master (Roche, Konzern-Hauptsitz, Grenzacherstrasse, Switzerland). Expression value was normalized to GAPDH in the same sample and then normalized to the control. The sequences of the primer pairs are listed as followed: interleukin-6 (IL-6): sense, 5′-TAGTCCTTCCTACCCCAATTTCC-3′ and antisense, 5′-TTGGTCCTTAGCCACTCCTTC-3′; IL-1β: sense, 5′-CTCGTGCTGTCGGACCCCAT-3′ and antisense, 5′- AGTGTTCGTCTCGTGTTCGGAC-3′; IL-18: sense, 5′- CAGGCCTGACATCTTCTGCAA-3′ and antisense, 5′- CTCCAGCATCAGGACAAAGAAAGCCG-3′; tumor necrosis factor-α (TNF-α): sense, 5′- AAGCCTGTAGCCCACGTCGTA-3′ and antisense, 5′- GGCACCACTAGTTGGTTGTCTTTG-3′; GAPDH: sense, 5′-GTATGACTCCACTCACGGCAAA-3′ and antisense, 5′- GGTCTCGCTCCTGGAAGATG-3′.

### Transmission Electron Microscopy

Murine BV2 microglia were cultured at 37°C on glass coverslips overnight, followed by the treatments mentioned above. BV2 microglia were harvested and fixed overnight at 4°C in 2.5% glutaraldehyde in 0.1 M PBS, and then post-fixed in 1% buffered osmium tetroxide for 2 h. Specimens were processed in routine procedure and examined under a transmission electron microscope (H-700; Hitachi, Tokyo, Japan).

### Autophagy Flux Assessment

Murine BV2 microglia were seeded on the cultural slides and transfected with tandem fluorescent mRFP-GFP-LC3 plasmid (HanBio, Shanghai, China) when the confluence reached to 50–70% (34, 35). In brief, after the culture in DMEM supplemented with 10% (vol/vol) FBS for 24 h, cells were incubated with plasmids for 6 h and then changed back to fresh DMEM supplemented with 10% (vol/vol) FBS for the cultivation of another 36 h to ensure the expression of the genes. After transfection, cells were challenged with 100 ng/ml LPS for 12 h in the presence or absence of PNU282987 (10 µM) for 10 min. Cellular autophagosomes (G^+^R^+^) and autolysosomes (G^−^R^+^) were detected by confocal microscopy (Leica TCS SP8, Leica, Biberach, Germany). Total number of puncta (>1 μm) per cell was counted.

### Cell Viability Assay

Cell viability was evaluated using a non-radioactive cell counting kit-8 (CCK-8; Dojindo, Kamimashiki-gun Kumamoto, Japan) as described previously (36). In brief, murine BV2 microglia at the density of 1 × 10^4^ were seeded in a 96-well plate in DMEM supplemented with 10% (vol/vol) FBS. After cultivation for 6 h, cells were treated as described above. After 12 h treatment, CCK-8 culture medium was added for 1 h additional cultivation. Absorbance was assayed with a microplate reader (Tecan Group Ltd., Männedorf, Switzerland) at the wavelength of 450 nm for the analysis of cell viability.

### Statistics

Data were presented as mean ± SEM. A two-way analysis of variance (ANOVA) followed by Bonferroni *post hoc* test for repeated measures was used for the analysis of the statistical significance of the EAE clinical scores between treatments. For other analysis, a Kruskal–Wallis test followed by Dunn’s *post hoc* test and one-way ANOVA followed by Bonferroni *post hoc* test were used to determine non-parametric data and continuous variables, respectively. A *P*-value <0.05 was considered statistically significant. Data were analyzed with SPSS 21.0K for Windows (SPSS, Chicago, IL, USA).

## Results

### Activating α7nAChR Ameliorates EAE Severity and Spinal Inflammatory Infiltration in EAE

**Figure 1:**
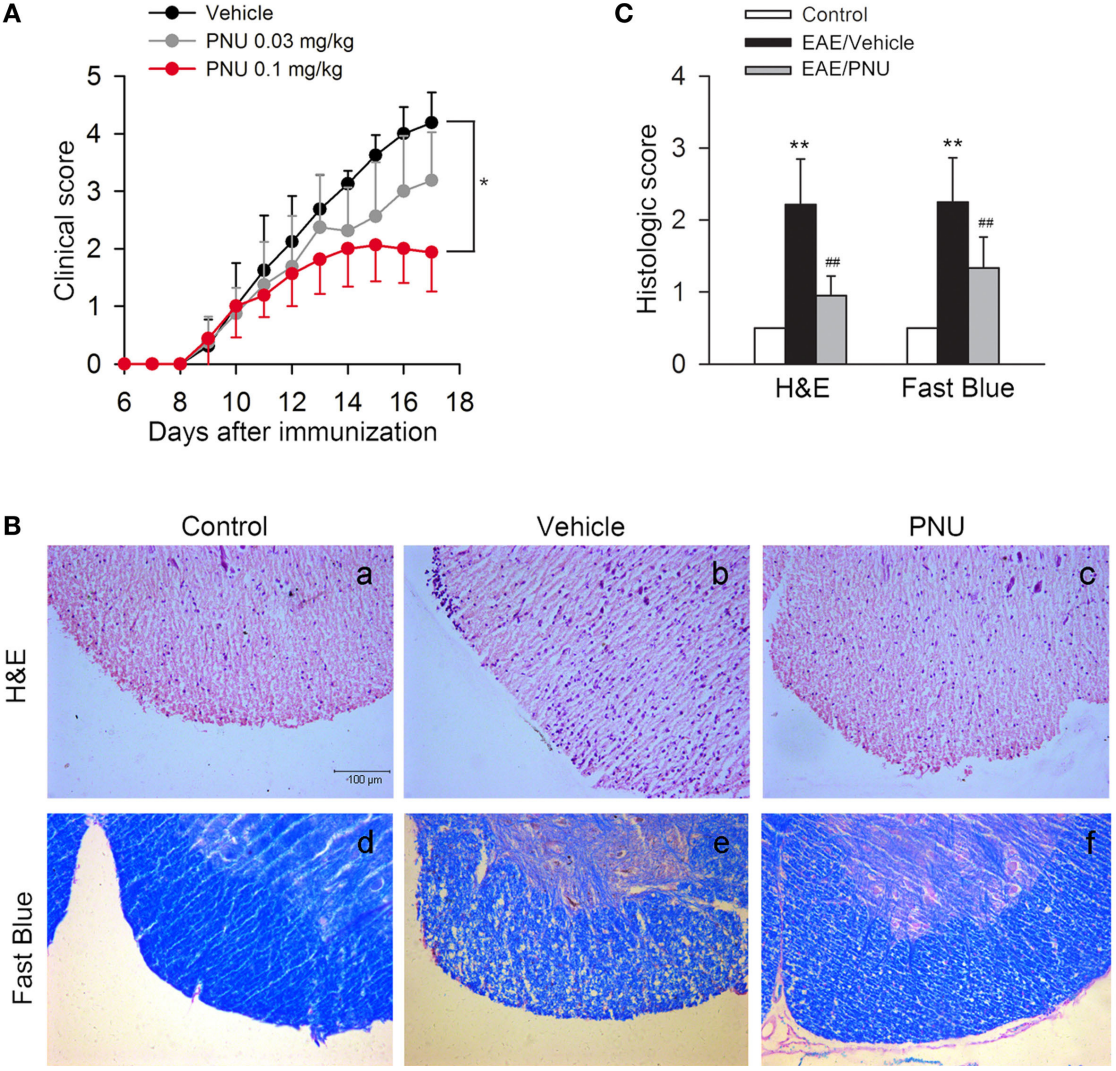
**α7nAChR activation alleviates experimental autoimmune encephalomyelitis (EAE) severity and central nervous system inflammatory infiltration in EAE mice**. EAE mouse model was created and treated with PNU282987 (0.03 and 0.1 mg/kg, i.p.) or vehicle from day 3 postimmunization and were maintained on drug for the duration of the study. **(A)** Clinical signs were assessed daily. 0.1 mg/kg PNU282987 effectively reduced the peak severity and cumulative clinical score of EAE mice while only slight decreases in the peak severity and cumulative clinical score were detected at the dose of 0.03 mg/kg body weight (*n* = 8 per group). *P < 0.05 vs vehicle. **(B,C)** H&E and luxol fast blue staining of paraffin sections of spinal cords isolated from control, vehicle, or PNU282987 (0.1 mg/kg)-treated EAE mice on day 17 (*n* = 8 per group). PNU282987 ameliorated spinal inflammatory infiltration and demyelination compared with vehicle. Scale bar, 100 µm. **P < 0.01 vs control; ^##^*P* < 0.01 vs vehicle. Veh, vehicle; PNU, PNU282987.

To assess the role of α7nAChR on CNS disorders, we treated EAE mice with α7nAChR agonist PNU282987 in the doses of 0.03 and 0.1 mg/kg body weight, respectively, from days 3 postimmunization (PI) till the end of study, and found that PNU282987 at the dose of 0.1 mg/kg body weight could effectively reduce the peak severity and cumulative clinical score of EAE while only slight decreases in the peak severity and cumulative clinical score were detected at the dose of 0.03 mg/kg body weight (Figure 1A). Histological study of spinal cord was performed at day 17 PI. Compared with vehicle, PNU282987 (0.1 mg/kg) caused a significant reduction of leukocyte infiltration and alleviative demyelination in spinal cord shown by H&E and luxol fast blue staining (Figures 1B,C).

### Activating α7nAChR Increases Autophagy in Spinal Cord and Spleen from EAE Mice

**Figure 2:**
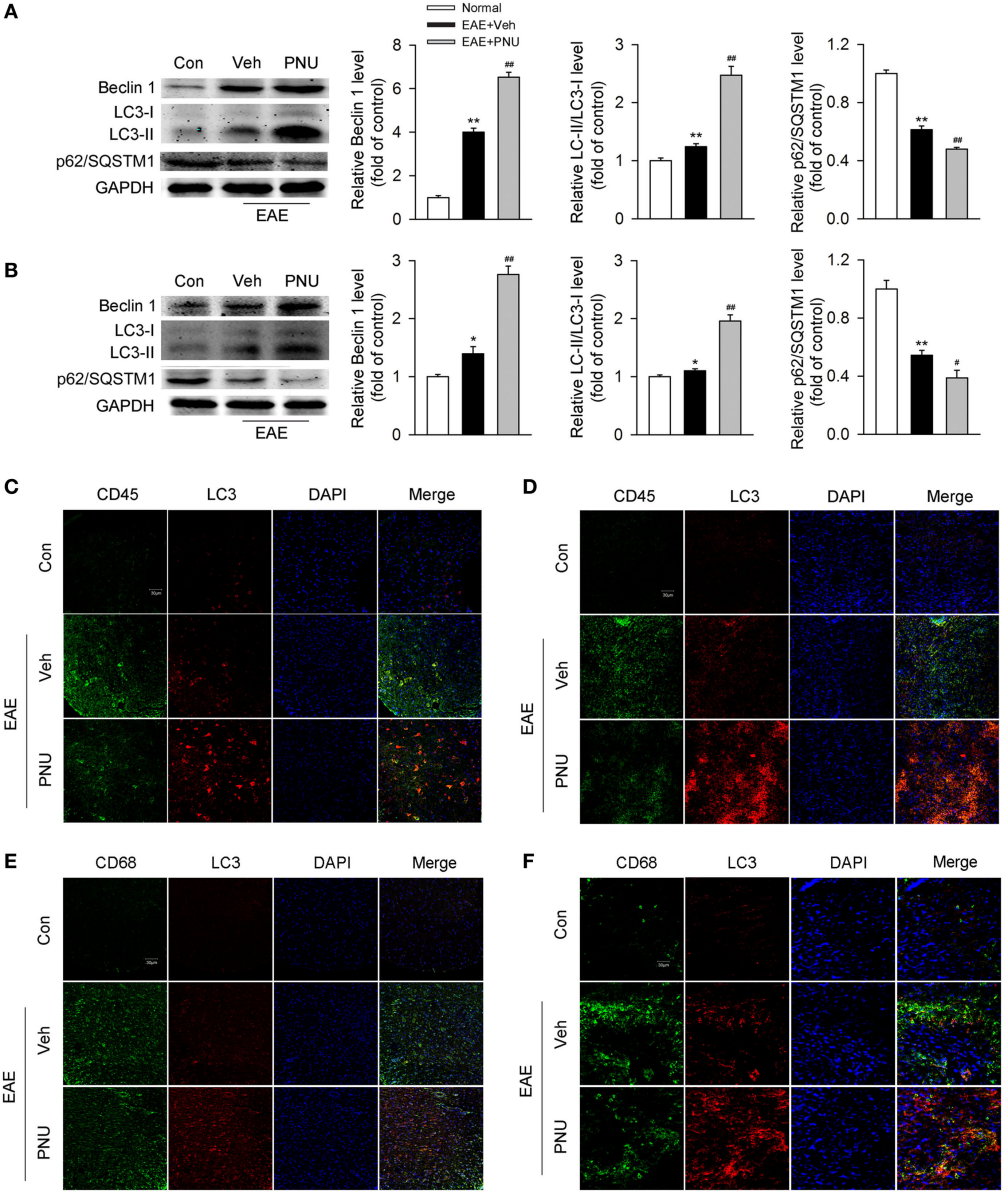
**α7nAChR activation leads to the enhancement of monocyte/macrophage autophagy in spinal cord and spleen from experimental autoimmune encephalomyelitis (EAE) mice**. EAE mouse model was created and treated with PNU282987 (0.1 mg/kg, i.p.) or vehicle from day 3 postimmunization (PI) and were maintained on drug for the duration of the study. **(A,B)** Spinal cord and spleen were isolated from mice on day 17 PI, and then lysed with buffer. **(A)** Levels of autophagy-related proteins were detected by Western blotting in spinal cord. PNU282987-treated EAE mice significantly enhanced the levels of Beclin 1 and LC3-II/I ratio and reduced the level of p62/SQSTM1 compared with vehicle (*n* = 6 per group). Quantitative analysis was conducted for the relative levels of Beclin 1, LC3-II/I ratio, and p62/SQSTM1. **p < 0.01 vs Veh. **(B)** The same detections were conducted for the levels in spleen (*n* = 6 per group). *P < 0.05 vs control; **P < 0.01 vs control;^#^ *P* < 0.05 vs vehicle; ^##^*P* < 0.01 vs vehicle. **(C)** Representative images of immunofluorescence microscopy of spinal cord paraffin-embedded sections for the staining of CD45, LC3, and DAPI. Scale bar, 30 µm. **(D)** Representative images of immunofluorescence microscopy of spleen paraffin-embedded sections for the staining of CD45, LC3, and DAPI. Scale bar, 30 µm. **(E)** Representative images of immunofluorescence microscopy of spinal cord paraffin-embedded sections for the staining of CD68, LC3, and DAPI. Scale bar, 30 µm. **(F)** Representative images of immunofluorescence microscopy of spleen paraffin-embedded sections for the staining of CD68, LC3, and DAPI. Scale bar, 30 µm. Veh, vehicle; PNU, PNU282987.

It has been previously demonstrated that autophagy plays a protective and ameliorative role in EAE through the regulation of inflammatory cytokine production (21). We thus ask whether the protective effect of activating α7nAChR on EAE is associated with the augmented autophagy. First, we tested the effect of activating α7nAChR on the levels of autophagy-related proteins in spinal cord and spleen from EAE mice and found that activating α7nAChR with PNU282987 significantly increased the LC3-II/I ratio and Beclin 1 abundance and decreased the p62/SQSTM1 abundance in spinal cord and spleen from EAE mice (Figures 2A,B).

Second, to determine the change of monocyte autophagy including macrophages in periphery and microglia in central (hereafter referred to as “monocyte/microglia autophagy”) upon activation of α7nAChR, we tested the colocalization of CD45- or CD68-positive cells with LC3, respectively, in spinal cord and spleen from the control and EAE mice with or without the treatment of PNU282987. We found that compared with the control group, EAE mice had more colocalization of CD45- or CD68-positive cells with LC3 in spinal cord and spleen. PNU282987 treatment further increased the colocalization of CD45 (Figures 2C,D) or CD68 (Figures 2E,F) with LC3, suggesting that activating α7nAChR contributed to the enhancement of monocyte/microglia autophagy in spinal cord and spleen from EAE mice.

### Blockade of Autophagy Attenuates the Protective Effects of α7nAChR Activation on EAE Mice

**Figure 3:**
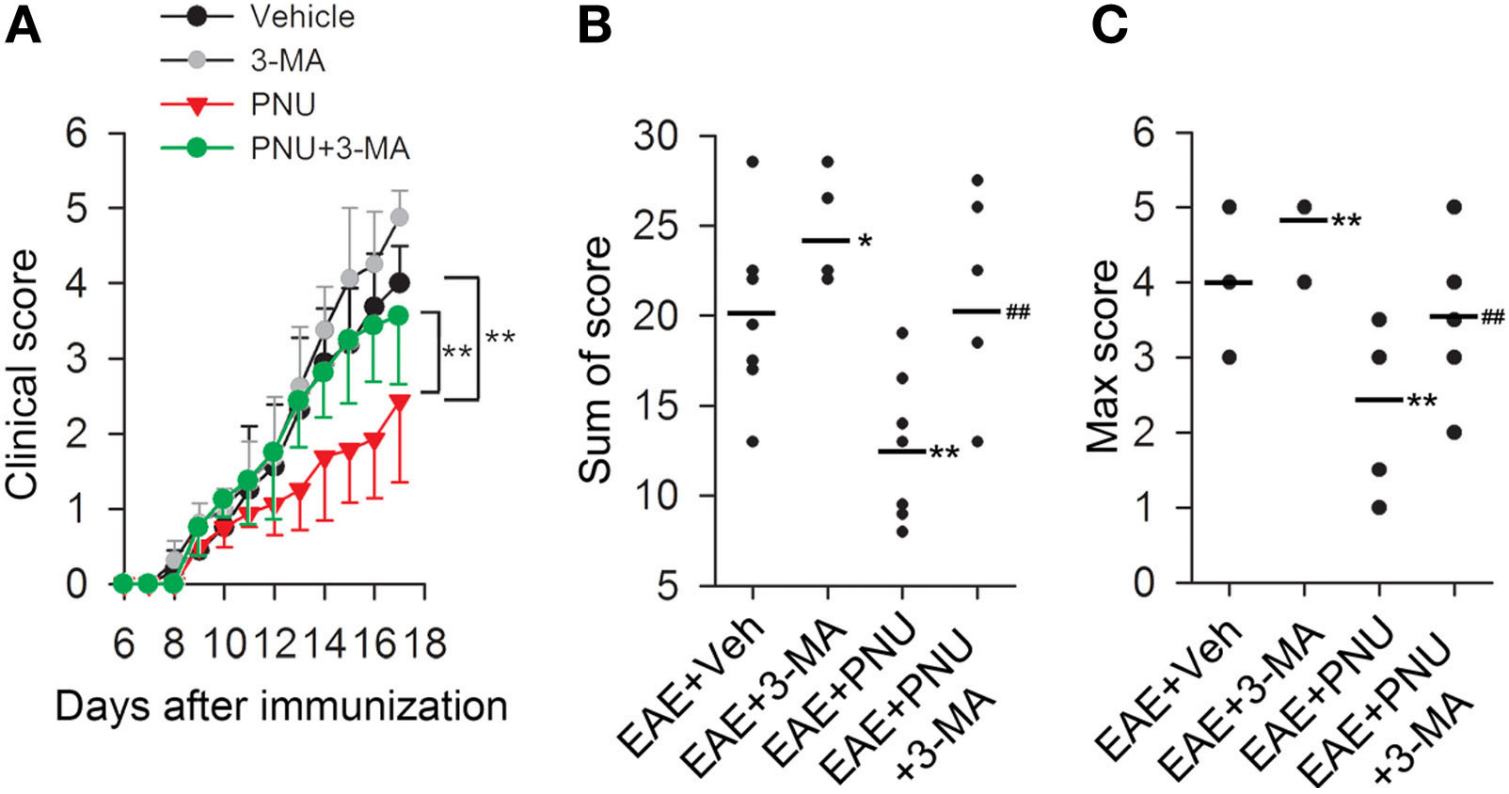
**Blockade of autophagy with 3-methyladenine (3-MA) attenuates the alleviative effects of α7nAChR activation on severity in experimental autoimmune encephalomyelitis (EAE) mice**. EAE mouse models were created. PNU282987 (0.1 mg/kg, i.p.) was given for the activation of α7nAChR and 3-MA (10 mg/kg, i.p.) was given for the blockade of autophagy process *in vivo*. The clinical score **(A)**, sum of score **(B)**, and max score **(C)** were assessed for the analysis of the extent of EAE severity (*n* = 8 per group). *P < 0.05 vs Veh, **P < 0.01 vs Veh, ^##^*P* < 0.01 vs PNU. Veh, vehicle; PNU, PNU282987.

To determine the influence of autophagy on the α7nAChR-mediated protective effects on the MS course *in vivo*, we examined neurobehavioral deficits in 3-MA, PNU282987, 3-MA + PNU282987, and vehicle-treated mice following MOG_35–55_-induction of EAE. As shown in Figure 1, the disease course in the EAE mice model was a chronic progressive-relapsing phenotype. PNU282987 (0.1 mg/kg, i.p.) treatment significantly reduced the severity of neurobehavioral deficits, cumulative scores, and maximum neurological disability in EAE mice compared with these vehicle-treated mice. This protective effect of PNU282987 was abolished by 3-MA (10 mg/kg), an autophagy inhibitor (Figures 3A–C), suggesting that autophagy at least partly played a role in the protective effects of activating α7nAChR on EAE.

**Figure 4:**
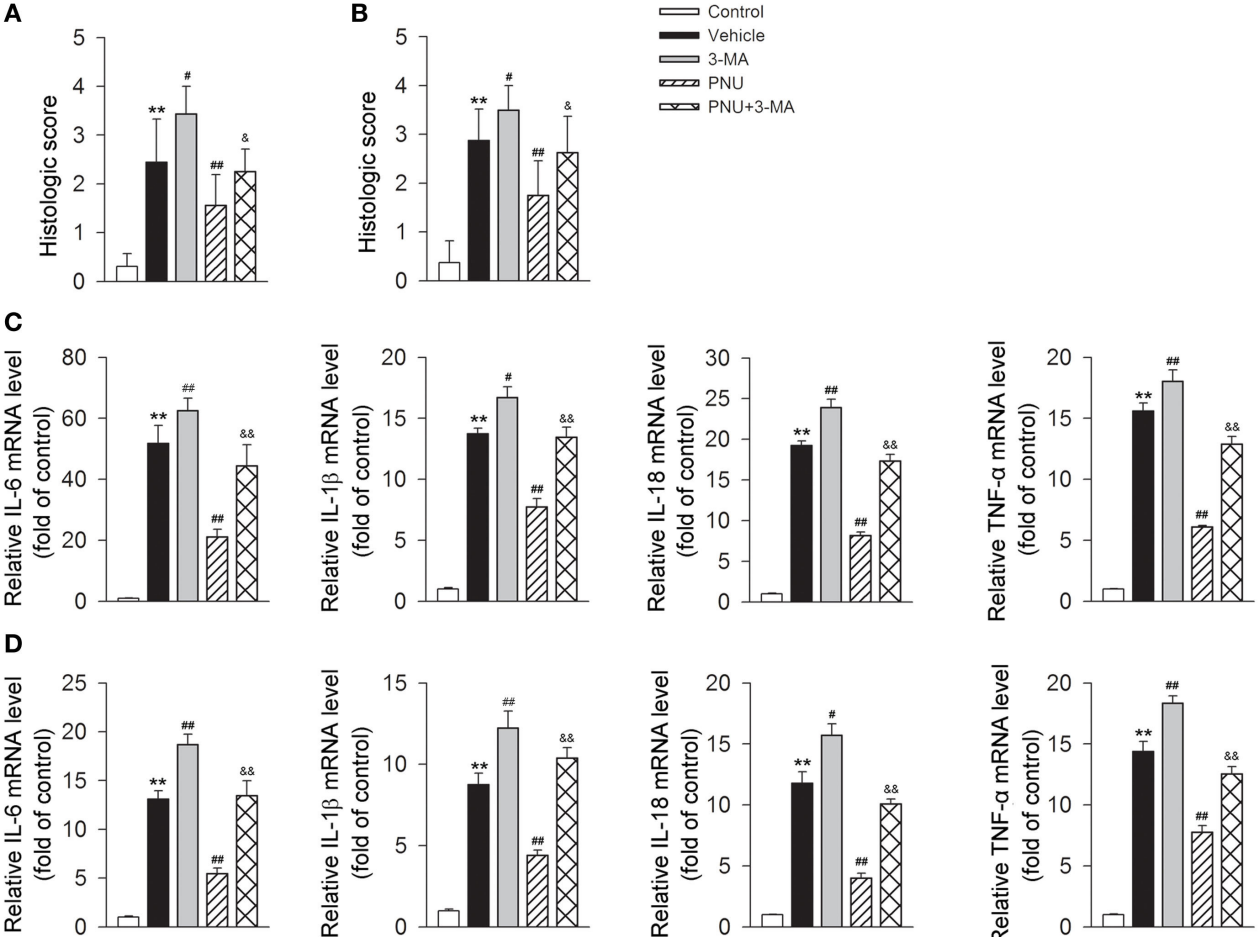
**Blockade of autophagy using 3-methyladenine (3-MA) attenuates the alleviative effects of α7nAChR activation on severity and inflammatory response in experimental autoimmune encephalomyelitis (EAE) mice**. EAE mouse model was created. PNU282987 (0.1 mg/kg, i.p.) was given for the activation of α7nAChR and 3-MA (10 mg/kg, i.p.) was given for the blockade of autophagy process *in vivo*. Spinal cord and spleen were isolated from mice on day 17 postimmunization. **(A)** Quantitative analysis of hematoxylin and eosin staining for the testing of the extent of spinal inflammatory infiltration (*n* = 8 per group). **P < 0.01 vs control, ^#^*P* < 0.05 vs Veh,^##^ *P* < 0.01 vs Veh,^&^ *P* < 0.05 vs PNU. **(B)** Quantitative analysis of luxol fast blue staining for the testing of the extent of spinal demyelination (*n* = 8 per group). **P < 0.01 vs control, ^##^*P* < 0.01 vs Veh, ^##^*P* < 0.01 vs Veh,^&^ *P* < 0.05 vs PNU. Production of IL-6, IL-1β, IL-18, and tumor necrosis factor-α (TNF-α) in spinal cord **(C)** and spleen tissue **(D)** in mRNA level was detected by real-time PCR. The blockade of autophagy process with 3-MA significantly increased the production of IL-6, IL-1β, IL-18, and TNF-α in mRNA level (*n* = 6 per group). **P < 0.01 vs control, ^#^*P* < 0.05 vs Veh, ^##^*P* < 0.01 vs Veh,^&&^ *P* < 0.01 vs PNU. Veh, vehicle; PNU, PNU282987.

As shown above, in EAE mice, the protective effects of PNU282987 treatment was associated with reduced inflammatory infiltration and demyelination. We then tested if 3-MA abolished the protective effects of PNU282987 by upregulating inflammatory infiltration and demyelination. We found that blockade of autophagy with 3-MA greatly attenuated the alleviative effects of PNU282987 on inflammatory infiltration and demyelination (Figures 4A,B). We further investigated the association between autophagy and α7nAChR-mediated anti-inflammatory effects *in vivo* by examining the production of IL-6, IL-1β, IL-18, and TNF-α in mRNA level in spinal cord and spleen obtained from mice. In EAE mouse model, the treatment of PNU282987 significantly decreased the mRNA levels of IL-6, IL-1β, IL-18, and TNF-α in spinal cord and spleen, while blockade of autophagy with 3-MA greatly abolished this effect (Figures 4C,D). Taken together, these data indicated that blockade of autophagy with 3-MA greatly attenuated the protective effect of α7nAChR activation on the alleviation of EAE symptoms and inhibition of inflammation in EAE mice.

### Activating α7nAChR Increases Autophagy in BV2 Microglia Stimulated with LPS

**Figure 5:**
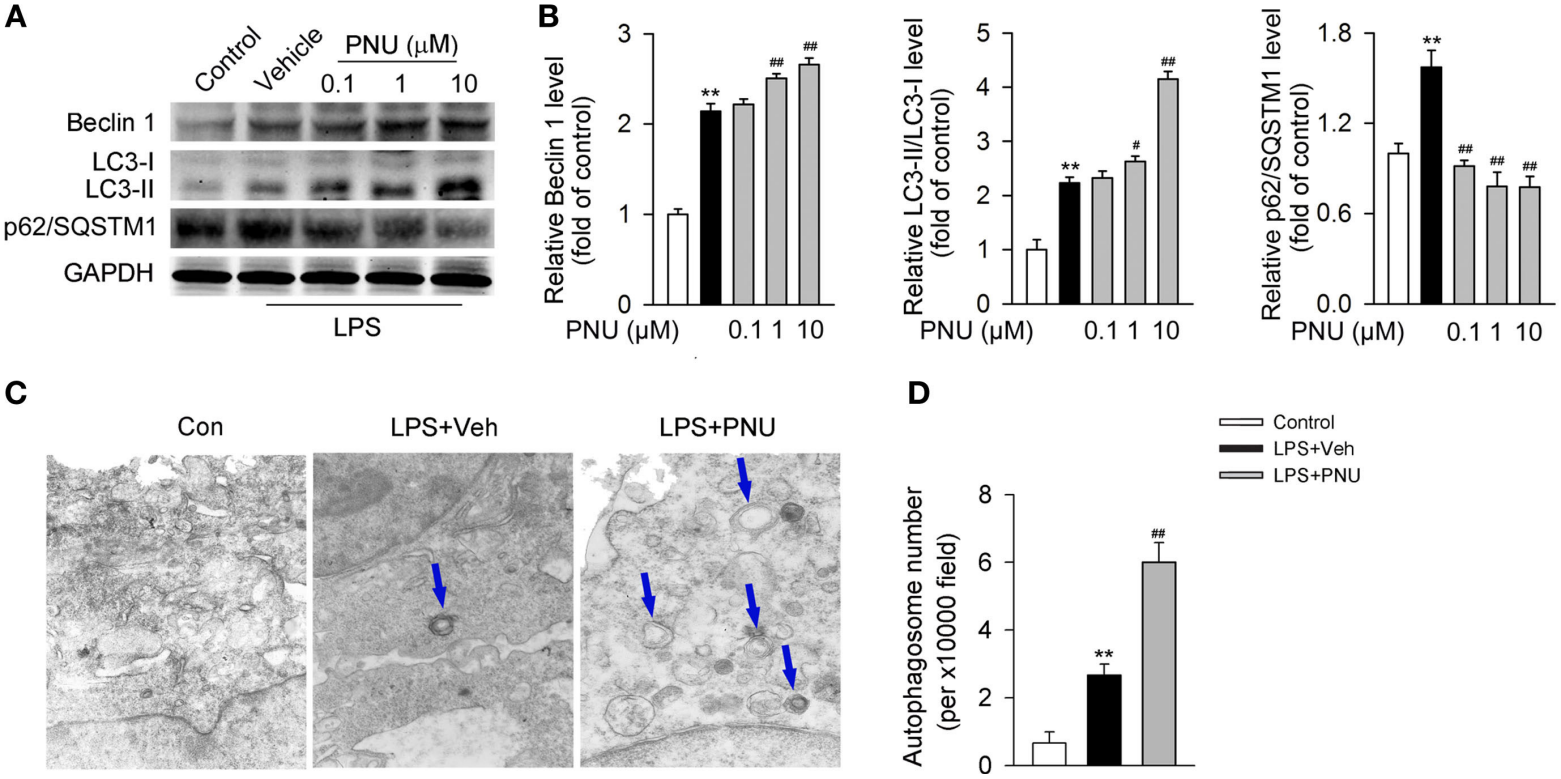
**α7nAChR activation leads to the enhancement of autophagy in BV2 microglia stimulated with lipopolysaccharide (LPS)**. **(A)** BV2 microglia were pretreated with vehicle or PNU282987 in three different doses (0.1, 1, and 10 µM) and then challenged with LPS (100 ng/ml). Relative expressions of autophagy-related proteins were detected by Western blotting. PNU282987 significantly enhanced the levels of Beclin 1 and LC3-II/I ratio and reduced the expression of p62/SQSTM1 compared with vehicle (*n* = 6 per group). **(B)** Quantitative analysis of relative levels of Beclin 1, LC3-II/I ratio, and p62/SQSTM1. **P < 0.01 vs normal, ^#^*P* < 0.05 vs Veh,^##^ *P* < 0.01 vs Veh. **(C)** BV2 microglia were pretreated with vehicle or PNU282987 (10 µM) and then challenged with LPS (100 ng/ml). The number of autophagosomes was detected by transmission electron microscopy and autophagosomes were marked by blue arrows. PNU282987 significantly increased the number of autophagosomes compared with vehicle (*n* = 6 per group). Magnification, 10,000×. **(D)** Quantitative analysis of the number of autophagosomes in BV2 microglia. **P < 0.01 vs normal, ^##^*P* < 0.01 vs Veh. Veh, vehicle; PNU, PNU282987.

Upon LPS stimulation, the levels of LC3-II/I ratio and Beclin 1 abundance were significantly increased in BV2 microglia. Whereas p62/SQSTM1, a cargo receptor targeting the substrates into forming autophagosomes, was significantly decreased (Figures 5A,B). Preincubation with PNU282987 (0.1, 1, and 10 µM) dose-dependently enhanced these LPS-induced effects in BV2 microglia (Figures 5A,B). Since autophagosomes are basic functional units for autophagy, here we further detected the number of autophagosomes *in vitro*. Under the stimulation of LPS, the number of autophagosomes was greatly increased which was detected by transmission electron microscopy. In addition, PNU282987 (10 µM) treatment further enhanced this effect (Figures 5C,D).

### Activating α7nAChR Promotes the Level of Autophagy Flux in BV2 Microglia Stimulated with LPS

**Figure 6:**
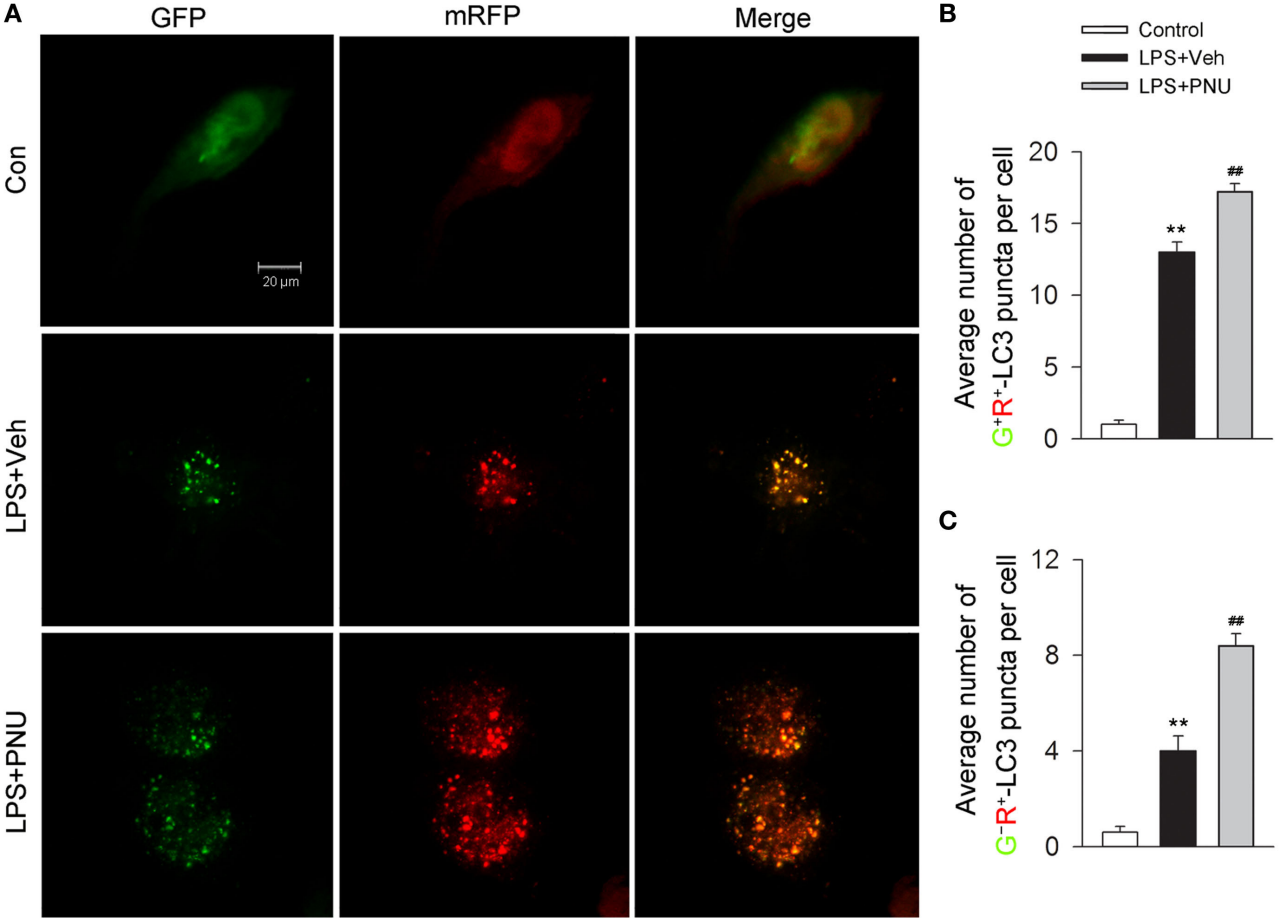
**α7nAChR activation increases the level of autophagy flux in BV2 microglia stimulated with lipopolysaccharide (LPS)**. **(A)** BV2 microglia were pretreated with vehicle or PNU282987 (10 µM) and representative images of mRFP-GFP-LC3 vector were shown by fluorescent detection. PNU282987 significantly increased the level of autophagy compared with vehicle (*n* = 5 per group). Scale bar, 20 µm. **(B,C)** Quantitative analysis of the number of yellow autophagosomes and red autolysosomes. **P < 0.01 vs normal, ^##^*P* < 0.01 vs Veh. Veh, vehicle; PNU, PNU282987.

Autophagy is considered as a recycling process that includes the maturation of autophagosomes and subsequently the fusion of autophagosomes and lysosomes for the formation of the degradative autolysosomes. Autophagy flux depicts this entire dynamic process. *In vitro*, the autophagy flux was previously reported to be detected by the transfection of adenovirus harboring mRFP-GFP-LC3 (35). After transfection, autophagosomes were shown as yellow punta (the combination of red and green fluorescence), and autolysosomes were shown as red punta (the extinction of GFP in the acid environment of lysosomes). As shown in Figures 6A–C, LPS challenge increased both the number of yellow autophagosomes and red autolysosomes (the extinction of GFP in the acid environment of lysosomes). Preincubation with PNU282987 further enhanced this effect induced by LPS, suggesting that activating α7nAChR increased the conversion from autophagosomes to autolysosomes and induced a high level of autophagy flux.

### Blockade of Autophagy by *Atg5* siRNA or Bafilomycin A1 Greatly Attenuates the Anti-inflammatory Effect of PNU282987 in BV2 Microglia Stimulated with LPS

**Figure 7:**
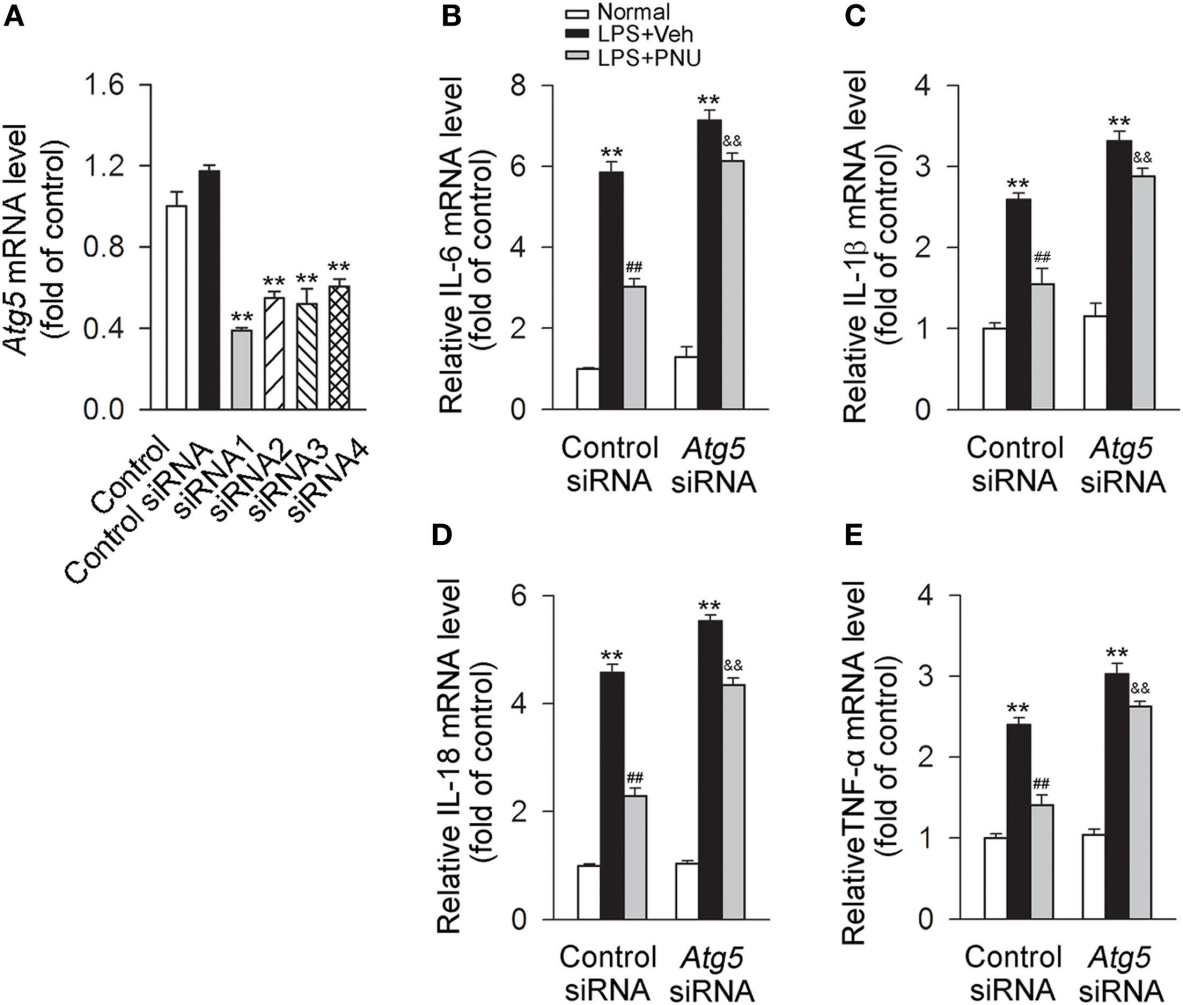
**Blockade of autophagy by *Atg5* siRNA greatly attenuates the anti-inflammatory effect of PNU282987 in BV2 microglia stimulated with lipopolysaccharide (LPS)**. **(A)** The quantitative expression of *Atg5* mRNA in BV2 microglia after transfection with the control siRNA or *Atg5* siRNAs. **P < 0.01 vs the control siRNA. After transfection with the control siRNA or *Atg5* siRNA [*Atg5* siRNA1 according to panel **(A)**], BV2 microglia were pretreated with vehicle or PNU282987 (10 µM) and then challenged with LPS (100 ng/ml). The control or *Atg5* siRNA were transfected (*n* = 6 per group). Production of interleukin-6 (IL-6)**(B)**, IL-1β **(C)**, IL-18 **(D)**, and tumor necrosis factor-α (TNF-α) **(E)** in mRNA level were detected by real-time PCR. The blockade of autophagy process with *Atg5* siRNA significantly increased the production of IL-6, IL-1β, IL-18 and TNF-α in mRNA level (*n* = 6 per group). **P < 0.01 vs normal, ^##^*P* < 0.01 vs Veh, ^&&^*P* < 0.01 vs control. Veh, vehicle; PNU, PNU282987.

Upon LPS stimulation, mRNA levels of IL-6, IL-1β, IL-18, and TNF-α in BV2 microglia were significantly increased. PNU282987 treatment significantly decreased the mRNA levels of inflammatory cytokines (IL-6, IL-1β, IL-18, and TNF-α). *Atg5*, an E3 ubiquitin ligase, is necessary for autophagy due to its role in autophagosome elongation. Knockdown of *Atg5* using *Atg5* siRNA (siRNA1 according to Figure 7A) significantly attenuated the inhibitory effect of PNU282987 on the levels of IL-6, IL-1β, IL-18, and TNF-α in mRNA (Figures 7B–E). Similar changes were found in the mRNA levels of IL-6, IL-1β, IL-18, and TNF-α with the application of bafilomycin A1 (Figure S1A in Supplementary Material). In addition, bafilomycin A1 also significantly inhibited the effect of PNU282987 on LC3-II/I ratio in LPS-stimulated BV2 microglia (Figures S1B,C in Supplementary Material). The administration of LPS, PNU282987, or bafilomycin A1 did not produce significant effect on cell viability (Figure S1D in Supplementary Material). Collectively, those results suggested that autophagy at least partly mediated the anti-inflammatory effect of PNU282987 in LPS-stimulated BV2 microglia.

### Participation of the AMPK–mTOR–p70S6K in the Protective Effects of PNU282987 in BV2 Microglia Stimulated with LPS

**Figure 8:**
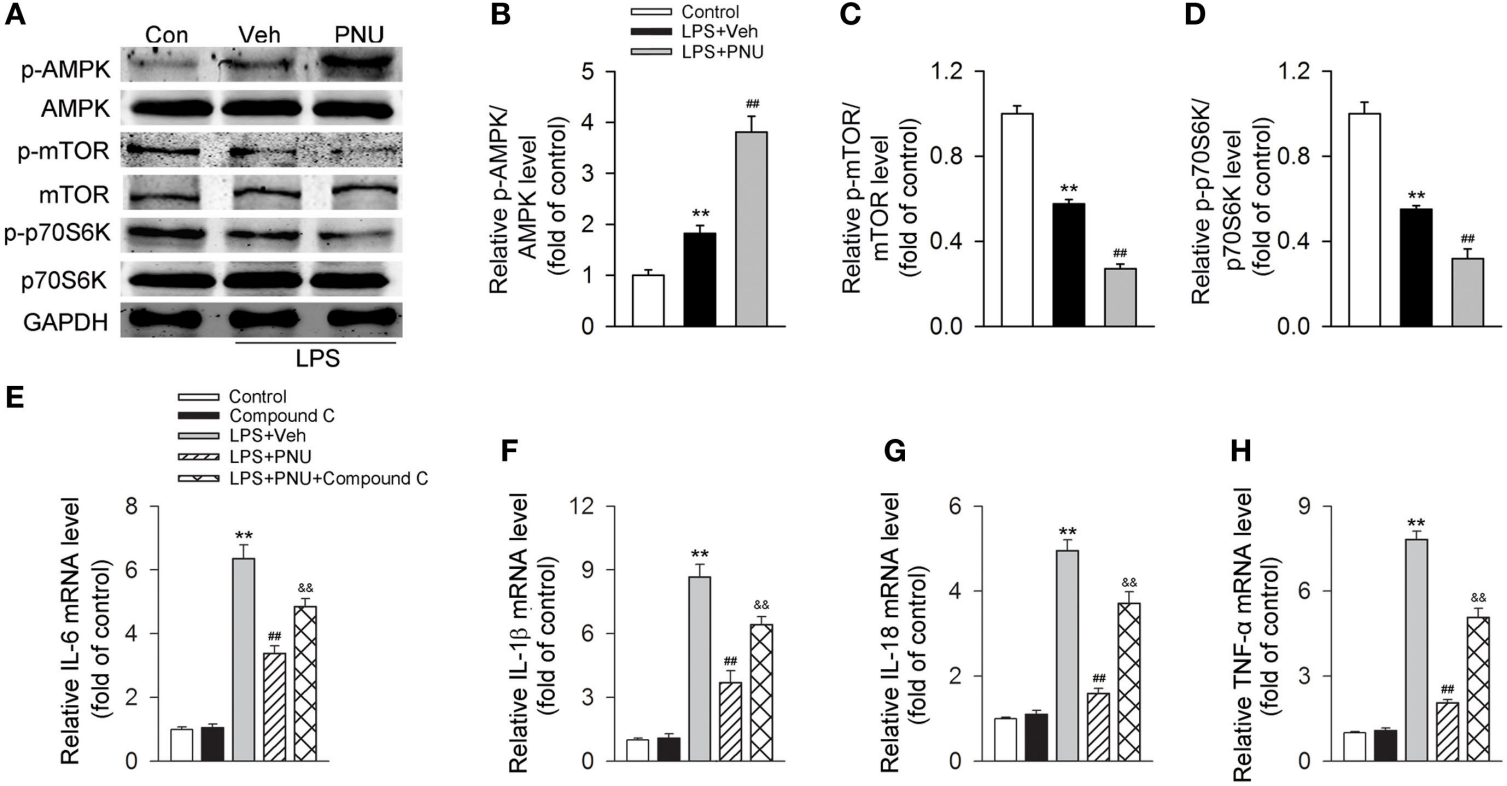
**Participation of the AMPK–mTOR–p70S6K axis in the anti-inflammatory effect of PNU282987 in BV2 microglia stimulated with lipopolysaccharide (LPS)**. BV2 microglia were pretreated with vehicle or PNU282987 (10 µM) and then challenged with LPS (100 ng/ml). **(A)** Levels of p-AMPK/AMPK, p-mTOR/mTOR, and p-p70S6K/p70S6K ratios were detected by Western blotting. PNU282987 significantly enhanced the ratios of p-AMPK/AMPK, p-mTOR/mTOR, and p-p70S6K/p70S6K compared with vehicle (*n* = 6 per group). **(B–D)** Quantitative analysis of relative levels of p-AMPK/AMPK, p-mTOR/mTOR, and p-p70S6K/p70S6K ratios. **P < 0.01 vs normal, ^##^*P* < 0.01 vs Veh. **(E–H)** Production of interleukin-6 (IL-6), IL-1β, IL-18, and tumor necrosis factor-α (TNF-α) in mRNA level were detected by real-time PCR. The blockade of the AMPK–mTOR–p70S6K signaling pathway with compound C (10 µM) significantly increased the production of IL-6, IL-1β, IL-18, and TNF-α in mRNA level (*n* = 6 per group). **P < 0.01 vs normal, ^##^*P* < 0.01 vs Veh. Veh, vehicle; PNU, PNU282987.

It has been previously demonstrated that AMPK activation inhibits mTOR and reduces p70S6K phosphorylation, thus promoting autophagy (37, 38). Here, we investigated whether the autophagy-inducing effect of activating α7nAChR was through the AMPK–mTOR–p70S6K signaling pathway in LPS-stimulated BV2 microglia. We found that LPS stimulation increased the phosphorylation of AMPK while decreased the phosphorylation of mTOR and p70S6K. PNU282987 treatment further increased the changes of phosphorylation of AMPK, mTOR, and p70S6K (Figures 8A–D). Furthermore, compound C (an AMPK inhibitor) significantly inhibited the PNU29287-mediated attenuation of IL-6, IL-1β, IL-18, and TNF-α production in LPS-stimulated BV2 microglia (Figures 8E–H). Taken together, these data indicated that the anti-inflammatory effects mediated by α7nAChR *via* inducing autophagy were at least partly through the AMPK–mTOR–p70S6K signaling pathway.

## Discussion

Our previous works have demonstrated that activating α7nAChR plays a protective role in several kinds of cardiovascular diseases including ischemic stroke, hypertension, and myocardial ischemia *via* the “cholinergic anti-inflammatory pathway.” It has been demonstrated previously that activating α7nAChR alleviates EAE through the inhibition of inflammatory reaction (14–16). For example, Hao et al. (16) demonstrated that α7nAChR was a key mediator in the process of nicotine-mediated reduction of the CNS inflammatory response and protection against EAE through the inhibition of auto-reactive T-cell proliferation and cytokine production from helper T cell. Consistent with those reports, in our present study, we demonstrated that activating α7nAChR significantly reduce the severity in clinical score and histological examination. The production of several inflammatory cytokines including IL-6, IL-1β, IL-18, and TNF-α was attenuated with the activation of α7nAChR in EAE mice. In combination, those data indicate that activating α7nAChR contributes greatly to the alleviation of EAE.

Autophagy has been recognized as a self-protective mechanism through degrading and recycling long-lived proteins, damaged organelles and misfolded proteins (39). Previous studies demonstrated that autophagy played an important role in neuroprotection through the modulation of inflammatory or immune reaction in CNS in MS or EAE (6, 40–43). For instance, it has been demonstrated (42) that the mammalian target of rapamycin (mTOR), a negative regulator of autophagy, was involved in microglial pro-inflammatory activation. Rapamycin, an mTOR inhibitor, ameliorated the clinical course of the relapsing-remitting as well as the chronic EAE through the induction of microglia autophagy. Autophagy level has also been demonstrated to be enhanced in EAE mice (34, 42). In our previous work, we demonstrated that activation of cannabinoid receptor 2 could induce autophagy, which subsequently led to the inhibition of NLRP3 inflammasome in CNS in EAE as well as colitis mice models (6, 44). Here, we reported that blocking autophagy with 3-MA could attenuate the anti-inflammatory and alleviative effects mediated by α7nAChR activation in EAE mice. Similar results were found in LPS-stimulated BV2 microglia when transfected with *Atg5* siRNA or the application of bafilomycin A1. Taken together, those data indicate that autophagy plays an important role in the amelioration of EAE as well as the anti-inflammatory effects in LPS-challenged BV2 microglia mediated by the activation of α7nAChR.

Recently, activating α7nAChR has been reported to play a protective role in CNS inflammation. The association between activating α7nAChR and induction of autophagy in neurons has been demonstrated. Jeong and Park reported that melatonin, a secretory hormone produced from various organs, enhanced the activation of autophagy process in neurons through the upregulation of α7nAChR signaling pathway, thus playing an important role in neuroprotection in prion-mediated neurodegenerative diseases (45). In their other article, the authors showed that the activation of α7nAChR stimulated by cellular prion protein expression contributed to the induction of autophagy flux in neurons, which played a pivotal role in neuroprotection (46). Their studies suggest an important role of α7nAChR – induce autophagy, which play a protective role in neuroprotection. Here, in our work, we demonstrated that activating α7nAChR contributed to the enhancement of autophagy both in spinal cord and spleen from EAE mice and BV2 microglia stimulated with LPS, and blockade of autophagy greatly attenuated its anti-inflammatory effect. Collectively, we believe that activating α7nAChR may suppress the inflammatory reaction through the induction of monocyte/microglia autophagy, thus playing an important role in neuroprotection as well as alleviation of EAE. Together with the recently proved neuroprotective effects of α7nAChR-mediated autophagy in neurons, we believe that enhancing α7nAChR-mediated autophagy may probably serve as a potential and promising therapeutic strategy in the treatment of CNS diseases.

In the *in vivo* studies, we used 3-MA to inhibit autophagy process for the detection of autophagy participation in the pathogenesis and progression of EAE, since 3-MA was widely used as an autophagy blocker (20, 47). Our results showed that 3-MA significantly attenuated the anti-inflammatory effects of activating α7nAChR in EAE model, thus demonstrating the involvement of α7nAChR-mediated autophagy in EAE disease. However, as discussed previously, the application of 3-MA as an autophagy blocker was not without its problems. It was reported that 3-MA could inhibit all clarifications of PtdIns3K and the related downstream signaling cascades, indicating that its specificity as an autophagy suppressor should be carefully considered (6, 20). It was further noted that the long-term application of 3-MA might slightly enhance autophagy level since 3-MA was not a satisfactory autophagy-specific inhibitor (48). In consideration of those limitations of 3-MA, *Atg5* siRNA was applied in the present study, since *Atg5* was regarded as a critical and necessary autophagy-related gene, participating the formation of autophagosome (49). We found that *Atg5* siRNA or the application of bafilomycin A1 significantly attenuated the inhibitory effect of PNU282987 on the mRNA levels of IL-6, IL-1β, IL-18, and TNF-α in BV2 microglia, indicating that autophagy at least partly mediated the anti-inflammatory effects of PNU282987 in LPS-stimulated BV2 microglia. According to those discussions, to further clarify the exact role of monoctye/microglia autophagy in EAE, other different kinds of autophagy inhibitors are demanded and more detailed works are warranted.

In this study, we demonstrated that the induction of autophagy by activating α7nAChR could produce the anti-inflammatory effects in microglia, which were at least partly through the AMPK–mTOR–p70S6K signaling pathway. However, previous studies have shown other signaling cascades involved in the α7nACh-mediated anti-inflammation (50–54). For example, it was reported that α7nAChR could regulate the immune and inflammatory reaction *via* PI3K–Akt–mTOR signaling cascade (52). Studies from our lab and others that JAK2–STAT3 cascade was involved in the anti-inflammatory effect induced by α7nAChR (53, 54). In addition, p38/Src-dependent pathway was considered to participate in the α7nAChR-mediated anti-inflammatory effects (50, 51). Based on those studies, we believe that the anti-inflammatory effects mediated by α7nAChR probably involve several signaling pathways. Together with these signaling cascades, autophagy, the already proved inflammatory suppressor, might jointly contribute to α7nAChR-mediated anti-neuroinflammation. Further studies need to be done for exploring their potential interaction.

## Conclusion

Taken together, we demonstrated the α7nAChR-mediated protective roles against neuroinflammation *via* AMPK–mTOR–p70S6K related autophagy in monocyte/microglia (Figure S2 in Supplementary Material). We first reported that activating α7nAChR contributed to the alleviation of EAE in severity and inflammatory infiltration. Furthermore, activating α7nAChR significantly led to the induction of autophagy both in spinal cord and spleen from EAE mice and LPS-challenged BV2 microglia. In addition, blockade of autophagy greatly attenuated the α7nAChR-mediated anti-inflammatory effects both in EAE mice and BV2 microglia stimulated with LPS. Finally, we reported that this process involved the AMPK–mTOR–p70S6K signaling pathway. These results demonstrated a novel mechanism for α7nAChR in MS or EAE, which might provide a potential therapeutic target in the treatment of MS and even other inflammation- or autoimmune-related disorders.

## Ethics Statement

This study was carried out in accordance with the recommendations of the guidelines of the Animal Care Committee of the Second Military Medical University, Shanghai, China. The protocol was approved by the Animal Care Committee of the Second Military Medical University.

## Author Contributions

B-ZS and PK conducted all experiments in animals and analyzed the data; Z-QX, B-ZH, and M-HC conducted experiments in cells; CL and B-ZS designed the study and wrote the manuscript; WW, X-WC, and D-FS revised the manuscript.

## Conflict of Interest Statement

The authors declare that the research was conducted in the absence of any commercial or financial relationships that could be construed as a potential conflict of interest.

## Supplementary Material

Figure S1 **Blockade of autophagy by bafilomycin A1 attenuates the anti-inflammatory effect of PNU282987 in BV2 microglia stimulated with lipopolysaccharide (LPS)**. BV2 microglia were treated with bafilomycin A1 (5 nM) for 10 min before the stimulation of PNU282987 (10 µM) and LPS (100 ng/ml) for 12 h. **(A)** Production of IL-6, IL-1β, IL-18, and tumor necrosis factor-α (TNF-α) in mRNA level were detected by real-time PCR. The blockade of autophagy process with bafilomycin A1 significantly increased the production of IL-6, IL-1β, IL-18, and TNF-α in mRNA level under the stimulation of LPS (*n* = 6 per group). **(B)** Expressions of LC3-I and LC3-II in protein were detected by Western blotting. Bafilomycin A1 significantly inhibited the effect of PNU282987 on LC3-II/I ratio in LPS-stimulated BV2 microglia (*n* = 6 per group).**(C)** Quantitative analysis of relative level of LC3-II/I ratio. **(D)** Cell viability was assessed by CCK-8 assays. The administration of LPS, PNU282987, or bafilomycin A1 did not produce significant effect on cell viability (*n* = 6 per group). ***P* < 0.01 vs normal, ^##^*P* < 0.01 vs Veh, ^&&^*P* < 0.01 vs control. Veh, vehicle; PNU, PNU282987; bafilomycin A1, Baf A1.

Figure S2 **Schematic illustration of the protective mechanism of α7nAChR activation through the induction of autophagy in monocyte/microglia**. **(A)** Under the occurrence of experimental autoimmune encephalomyelitis (EAE) or the stimulation of lipopolysaccharide (LPS), inflammatory response is greatly triggered in monocyte/microglia which significantly overwhelms the induced protective autophagy, thus leading to the deterioration of EAE severity. **(B)** However, activating α7nAChR by PNU282987, a specific α7nAChR agonist, largely increased the level of autophagy in monocyte/microglia, which contributes to the inhibition of inflammation and consequently the alleviation of EAE, restoring the balance between the protective autophagy and inflammation. PNU, PNU282987.
