# Supplementary figures and images for: Autophagy Plays an Important Role in Anti-inflammatory Mechanisms Stimulated by Alpha7 Nicotinic Acetylcholine Receptor
Source: Front Immunol. 2017 May 16;8:553. doi: 10.3389/fimmu.2017.00553 (PMC5432615; doi:10.3389/fimmu.2017.00553)

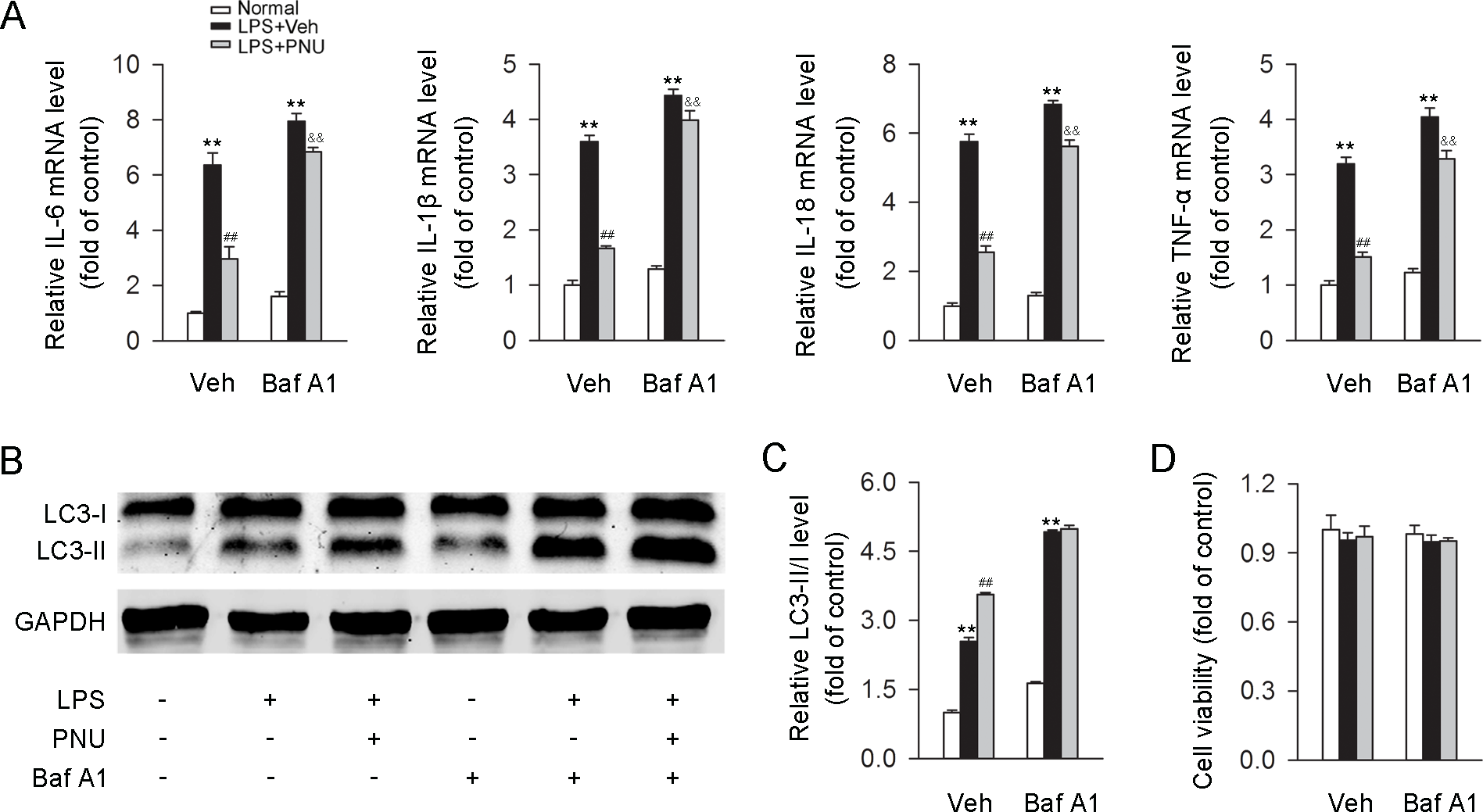

Supplement: Figure S1 — Blockade of autophagy by bafilomycin A1 attenuates the anti-inflammatory effect of PNU282987 in BV2 microglia stimulated with lipopolysaccharide (LPS). BV2 microglia were treated with bafilomycin A1 (5 nM) for 10 min before the stimulation of PNU282987 (10 µM) and LPS (100 ng/ml) for 12 h. (A) Production of IL-6, IL-1β, IL-18, and tumor necrosis factor-α (TNF-α) in mRNA level were detected by real-time PCR. The blockade of autophagy process with bafilomycin A1 significantly increased the production of IL-6, IL-1β, IL-18, and TNF-α in mRNA level under the stimulation of LPS (n = 6 per group). (B) Expressions of LC3-I and LC3-II in protein were detected by Western blotting. Bafilomycin A1 significantly inhibited the effect of PNU282987 on LC3-II/I ratio in LPS-stimulated BV2 microglia (n = 6 per group).(C) Quantitative analysis of relative level of LC3-II/I ratio. (D) Cell viability was assessed by CCK-8 assays. The administration of LPS, PNU282987, or bafilomycin A1 did not produce significant effect on cell viability (n = 6 per group). **P < 0.01 vs normal, ##P < 0.01 vs Veh, &&P < 0.01 vs control. Veh, vehicle; PNU, PNU282987; bafilomycin A1, Baf A1. [file Image_1.TIF]

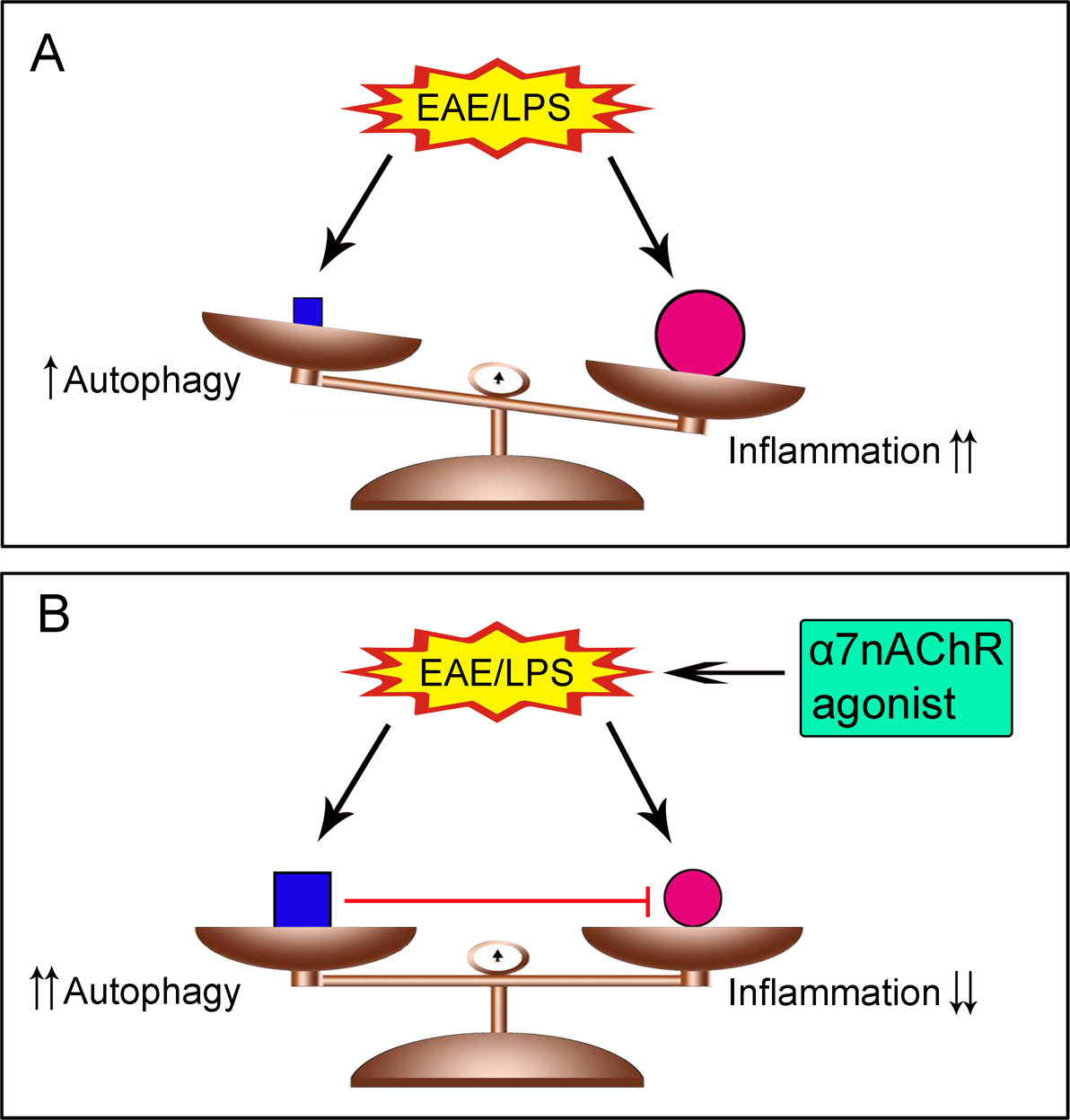

Supplement: Figure S2 — Schematic illustration of the protective mechanism of α7nAChR activation through the induction of autophagy in monocyte/microglia. (A) Under the occurrence of experimental autoimmune encephalomyelitis (EAE) or the stimulation of lipopolysaccharide (LPS), inflammatory response is greatly triggered in monocyte/microglia which significantly overwhelms the induced protective autophagy, thus leading to the deterioration of EAE severity. (B) However, activating α7nAChR by PNU282987, a specific α7nAChR agonist, largely increased the level of autophagy in monocyte/microglia, which contributes to the inhibition of inflammation and consequently the alleviation of EAE, restoring the balance between the protective autophagy and inflammation. PNU, PNU282987. [file Image_2.TIF]
